# Chirality-Induced Spin–Orbit Coupling and Spin Selectivity

**DOI:** 10.1021/acs.jpca.5c05383

**Published:** 2025-09-30

**Authors:** Massimiliano Di Ventra, Rafael Gutierrez, Gianaurelio Cuniberti

**Affiliations:** † Department of Physics, 8784 University of California San Diego, La Jolla, California 92093, United States; ‡ Institute for Materials Science and Max Bergmann Center of Biomaterials, 9169 TU Dresden, 01062 Dresden, Germany; § Dresden Center for Computational Materials Science (DCMS), TU Dresden, 01062 Dresden, Germany

## Abstract

We show that a spinor traveling along a one-dimensional helical path develops a spin–orbit coupling as a result of the curvature of the path. We estimate the magnitude of the associated spin polarization and obtain values typical of many helical molecular structures that showcase the Chirality-induced Spin Selectivity (CISS) effect. We find that this chirality-induced spin–orbit coupling (χ-SOC), in conjunction with broken time-reversal symmetry, may be an important ingredient for the microscopic underpinning of the CISS phenomenon.

## Graphical abstract

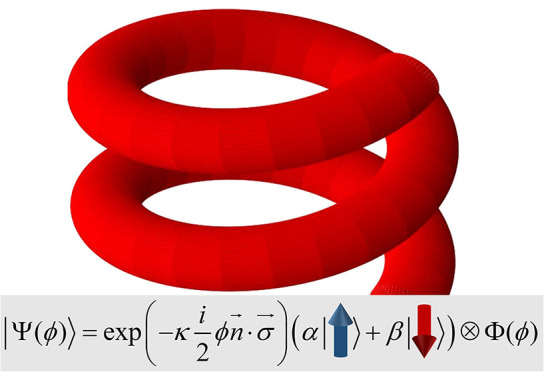

## Introduction

Spin–orbit coupling (SOC) is a fundamental relativistic phenomenon arising from the coupling between the spin and orbital degrees of freedom of a spinful particle.^1^ In atomic systems, SOC scales as *Z* ^4^ with *Z* being the atomic number; thus, SOC is typically smaller the lighter the atoms. The spin itself can also be manipulated by coupling it to a magnetic field. Therefore, it was a complete surprise that chiral molecules primarily made of organic elements, hence with weak SOC, display a spin response (selectivity) in the absence of any external magnetic field.^2–18^ This phenomenon has been called Chirality-induced Spin Selectivity (CISS), and it has triggered a large amount of research in physics, chemistry, and biology, also in view of the broad spectrum of potential applications it may offer.^19–29^

There is agreement that SOC must play a key role in determining the CISS effect^30–39^ as well as time-reversal symmetry breaking via an applied voltage in transport junctions or by environmental decoherence.^35,40–45^ However, while time-reversal symmetry breaking is relatively easy to account for, in view of the way experiments are performed, the origin of a non-negligible effective SOC has not yet been fully elucidated.^38,46–55^ Meanwhile, the coupling to vibrational degrees of freedom, leading to electron-vibration and spin-vibration coupling, has also been discussed.^49–51,56,57^

On the side of first-principle calculations, there is no full agreement concerning the orders of magnitude of the spin polarization,^42,58–62^ so that the ultimate origin of the CISS effect remains under debate. A computational study,^62^ based on a fully relativistic density functional theory method combined with the Landauer-Büttiker approach, has suggested the need to include geometric terms in the SOC to achieve closer agreement with experimental trends. Also, a recent investigation based on a time-dependent relativistic four-current framework suggests that CISS might be related to relativistic curvature-induced helical currents and the associated magnetic fields, equally pointing to the relevance of geometrical effects.^63^

It is therefore very appealing to see spin–orbit coupling emerging from a simple geometric principle. In refs 64–66 such a geometric SOC was derived. Shitade and Minamitani^64^ started from the Dirac Lagrangian density in a curved space-time to arrive at an SOC expression proportional to the curvature of a helix. This SOC includes the product of the projection of the Pauli spin vector σ⃗ in the direction of the helix binormal vector *B⃗* (using a Frenet-Serret basis), and the linear momentum *p*_*s*_ of the electron along the helix: (σ⃗ ·*B⃗*)*p*_*s*_. An estimate of the coupling strength was obtained to be approximately 160 meV, which is far stronger than any atomic SOC of light atoms. However, there seems to be a potential issue with the approach of Shitade and Minamitani, which the authors also acknowledge: the final results can be different according to whether the thin-layer quantization is performed before or after the Foldy-Wouthuysen transformation to obtain the nonrelativistic limit of the Dirac equation. In fact, Yu^65,66^ exploited the relativistic equivalence of a curved space-time manifold and a noninertial system to obtain a different result, in terms of the local normal vector *N⃗*: (*N⃗* × *p⃗*)·σ⃗. An estimate of the coupling constant yielded in this case 0.2 meV, but for a reference polymer system with a much larger radius and pitch than, e.g., DNA. We remark that, although the above difficulty does not appear when the Pauli equation^67,68^ or the spin-independent Schrödinger equation^69,70^ are taken as starting point, this does not solve the original problem, which so far has remained a not fully resolved mathematical issue.

Here we show, using a spin-independent Hamiltonian as a starting point, that the dynamics of a spinful particle along a helical path naturally develops a purely kinetic effective SOC, even if the particle does not experience any other potential (besides a spin-independent confinement potential transverse to a helical path). We find that this chirality-induced SOC, which we denote as χ-SOC, is substantial for systems that currently show the CISS effect. We suggest that, together with the breaking of time-reversal symmetry (originating, e.g., from the external bias applied in the experiments), this χ-SOC provides a simple way to generate a geometric SOC in chiral systems.

## Methodology: Effective 1d-Hamiltonian on a Helix

We consider the Hamiltonian of an electron on a curved path, in particular, on an infinite helical tube with finite cross section (see Figure 1). The Hamiltonian consists of a kinetic energy term *T̂* and a transverse confinement potential *V*, but no spin-dependent interactions are considered. When applied to real molecular systems, the confinement potential is related to the electrostatic potential distribution along the molecular frame, and it is, thus, dependent on the chemical composition. However, at the level of abstraction we are working, our choice is guided by Occam’s razor, so that we impose few minimal conditions: the confinement potential should (i) be a continuous function, (ii) have a minimum on all points along the helical pathway shown in Figure 1, (iii) allow for an analytically closed solution of the transverse Schrödinger equation, i.e., it should not depend on the arc length *s*, and (iv) be spin-independent.

**Figure 1:**
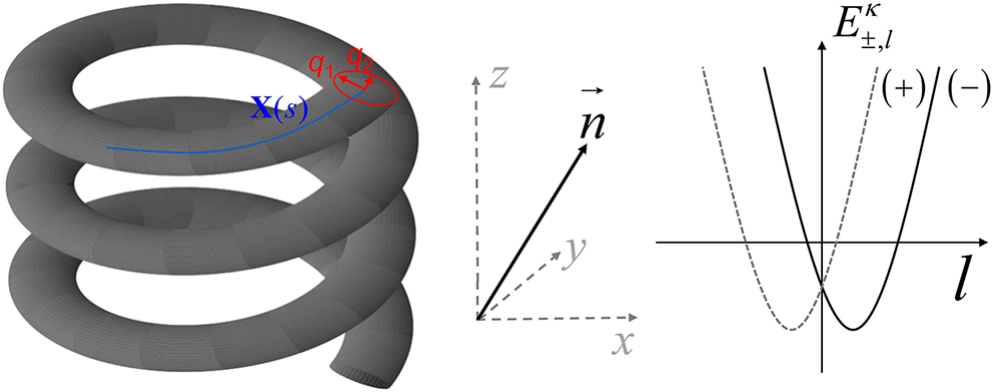
Schematic representation of the model system. Left panel: A helical tube is considered, which is subsequently mapped onto a one-dimensional helical path following the procedure described in the text. The tube can be parametrized by the helical path described by the vector **X**(*s*) with *s* being the arc length, and a pair of local transversal coordinates *q*_1_, *q*_2_. In the transverse direction (cross section), a generic confinement potential *V*_λ_(*q*_1_, *q*_2_) is assumed, where λ is a measure of the strength of the confinement, similar to a quantum well. Although the specific form of *V*_λ_(*q*_1_, *q*_2_) can be arbitrary, an SO(2) symmetric potential is assumed. Right panel: The eigenvalues of the Hamiltonian in [Disp-formula eq5] are schematically shown as a function of the angular momentum quantum number *l* for the two spin branches (“+” and “–”), cf. [Disp-formula eq8].

Following a procedure presented originally by da Costa,^69^ but also formalized by e.g., Maraner,^70^ and others (see e.g.,^71,72^ ), one can decouple longitudinal (along the helical path) and transverse degrees of freedom to map the 3d-Hamiltonian structure of the helical tube on an effective 1d-Hamiltonian. This procedure has, more recently, been formalized by Geyer et al.^37^ within a rigorous space-adiabatic framework,—a similar approach has also been used, e.g., in refs 39,67,73 . We skip here the details of the derivation, which are provided in the Supporting Information (SI) section for three different confinement potentials: (a) an SO(2) harmonic confinement, (b) square well harmonic confinement, and (c) a square well hard wall confinement. Here, we limit ourselves to present the results for the SO(2) symmetric potential.

The obtained effective 1d-Hamiltonian reads:

1 $${Ĥ}_{\mathrm{eff}}^{1d}=-\frac{\hbar^{2}}{2mL^{2}}\left\{\frac{\partial^{2}}{\partial \phi^{2}}+\frac{\rho R}{4}\right\}$$

where ρ = *R*/*L* ^2^ and τ = (*b*/2π)/*L* ^2^ are the curvature and torsion, respectively, of a helix with radius *R* and pitch *b*. $L=\sqrt{R^{2}+{\left(b/2\pi \right)}^{2}}$ is related to the length of a single helical turn *L*_0_ via *L* = *L*_0_/2π. The second term in eq 1 is a quantum geometric potential already obtained by da Costa,^69^ but also in other studies.^37,39,67,70,73^ Additional terms proportional to the torsion of the path may also appear for other choices of the confinement potential (see the SI section). However, they are all spin diagonal.

Consider now the general representation of a spinor wave function on the 1d helical path^74^ and with a Hamiltonian described by eq 1:

2 $$\mathrm{\Psi ⃗}\left(\phi \right)=\exp\left\{-i\kappa \frac{\phi }{2}\mathrm{n⃗}\cdot \mathrm{\sigma ⃗}\right\}\mathrm{\chi ⃗}⊗\Phi \left(\phi \right)=U\left(\phi ,\mathrm{n⃗}\right)\mathrm{\chi ⃗}⊗\Phi \left(\phi \right)$$

Due to the absence of SOC in eq 1 the spin and spatial components are separable. The 2-component spinor χ⃗ does not need to be specified at this stage, its components will be calculated later on. Notice that the spin rotation is tied to the space frame of the helix, which is also a result of the confinement potential used to enforce the electron to follow the helical pathway. In other words, the very presence of the helix constrains the electron to follow the curved path, with the spin following suit. The unitary operator acting on the spinor χ⃗ induces a spin rotation around *n⃗* while the electron moves along the helix (this is similar to the action of a quantum gate on a qubit, with the helix playing the role of the “quantum gate”). The parameter κ = ±1 accounts for a change from a right-handed to a left-handed helix, since the sign of ϕ changes in this case. Notice that the quantum geometric potential commutes with the spin rotation operator, and hence it will have no influence on the spin-dependent properties of the model.

The spatial part Φ­(ϕ) can be written as a linear combination of “plane wave” solutions with (real-valued) angular momentum *l* as

3 $$\Phi \left(\phi \right)=\int_{-\infty }^{\infty }\mathrm{dl}A_{l}e^{\mathrm{il}\phi }$$

Here, the normalization condition is ∫_0_^2π^ (*dϕ*/2π)|Φ­(ϕ)|^2^ = 1, from which it follows that ∫_–∞_^∞^ *dl*|*A*_*l*_|^2^ = 1. For the calculations of the charge and spin currents later on, it is convenient to restrict the integration to positive values of *l* by introducing the index *s* = sgn­(*l*) = ±1: *e* ^*ilϕ*^ → *e* ^*is*|*l*|ϕ^. Acting with the Hamiltonian eq 1 on the wave function eq 2, and defining *E*_0_ = ℏ^2^/2*mL* ^2^ as a characteristic energy scale of the problem, we obtain the following:

4 Ĥeff1dΨ⃗(ϕ)=E0U(ϕ,n⃗){(−i∂∂ϕ)2−κ(n⃗·σ⃗)(−i∂∂ϕ)+14(n⃗·σ⃗)2−ρR4}χ⃗⊗Φ(ϕ)=E0U(ϕ,n⃗){pϕ2−κ(n⃗·σ⃗)pϕ+(14−ρR4)}χ⃗⊗Φ(ϕ)

In the second equality, we have introduced the angular momentum operator *p*_ϕ_ = −*i*∂/∂ϕ and used the result (*n⃗* ·σ⃗)^2^ = 1. Therefore, we can introduce a new Hamiltonian as
5

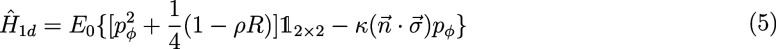

where we stress the fact that the kinetic energy operator and the correction leading to a geometric potential are both diagonal in spin space. These results show that it is possible to derive an effective spin–orbit coupling for an electron moving on a curvilinear path, even if no previous SOC was present. The key result is that the geometric phase accumulated by the spin during its motion leads to an effective interaction between the spin and the orbital degrees of freedom, which can be interpreted as a chirality-induced spin–orbit coupling term:

6 $$(\mathrm{L⃗}\cdot \mathrm{S⃗}{)}_{\chi -\mathrm{SOC}}\equiv \left(2E_{0}/\hbar \right)\kappa \left(\mathrm{n⃗}\cdot \mathrm{S⃗}\right)p_{\phi }$$

Notice that the obtained χ-SOC has a purely kinetic origin and its strength is controlled by the energy scale *E*_0_. For a DNA helix with *R* = 1 *nm* and *b* = 3.4 *nm*, one estimates *E*_0_ ≈ 30 *meV*, which is larger by a factor 3 to 4 than the atomic SOC of light elements.[Fn fna] The obtained geometric SOC is clearly time-reversal invariant, and it can also be rewritten as an SU(2) “pseudo-gauge field” by completing squares in eq 5:
7

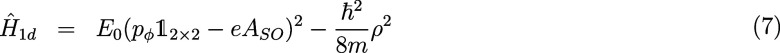

with $A_{\mathrm{SO}}=\frac{\kappa }{2e}\mathrm{n⃗}\cdot \mathrm{\sigma ⃗}$ . This approach leverages the properties of SU(2) rotations to capture the evolution of the spin state in a curved trajectory. Note that this contribution would also be present on a circle (*b* = 0), although in this case the angular momentum variable would be quantized due to the periodicity condition Φ­(ϕ + 2π) = Φ­(ϕ), but it would trivially vanish as *R* → ∞, i.e., in the limit of a straight line.

## Results and Discussion

The eigenvalues of eq 5 can be easily found:

8 $${Ẽ}_{\pm ,l}^{\kappa }=l^{2}+\frac{1}{4}\left(1-\rho R\right)\pm \kappa l={\left(l\pm \frac{\kappa }{2}\right)}^{2}-\frac{\rho R}{4}$$

where *Ẽ*_±,*l*_^κ^ = (*E*_±,*l*_^κ^)/*E*_0_. This represents two parabolas shifted horizontally from each other by κ (see schematic in Figure 1). The eigenvalues as a function of *l* yield two spin branches (“+” and “–”) for positive *l* (or *s* = 1), and another two spin branches for negative *l*. Due to time-reversal symmetry, the relation *Ẽ*_+,*l*_^κ^ = *Ẽ*_–,–*l*_^κ^ is valid, so that Kramer’s theorem holds, as expected.

The corresponding spinor eigenfunctions χ⃗ can be obtained in terms of the components of the vector *n⃗*, which we parametrize in general using two angles α, β as *n⃗* = (sin α cos β, sin α sin β, cos α). In this way, we get the following:

9 $${\mathrm{\chi ⃗}}_{+}=e^{i\beta /2}\left(\begin{array}{l} \sin\left(\alpha /2\right)e^{-i\beta /2}\\ -\cos\left(\alpha /2\right)e^{i\beta /2} \end{array}\right)$$

and

10 $${\mathrm{\chi ⃗}}_{-}=e^{-i\beta /2}\left(\begin{array}{l} \cos\left(\alpha /2\right)e^{-i\beta /2}\\ \sin\left(\alpha /2\right)e^{i\beta /2} \end{array}\right)$$

Another pair of eigenvectors is obtained for *s* = −1 simply by replacing *e* ^*is*|*l*|ϕ^ → *e* ^–*is*|*l*|ϕ^.

We can now calculate, in the local ϕ-frame, the average charge current *j*_*c*_^±^ and the average spin current *j*_spin_:

11 $$j_{c}^{\pm }=\int_{0}^{2\pi }\frac{d\phi }{2\pi }\;{\vec{\Xi }}_{\pm }^{†}\left(\phi \right)\left(e\hat{\nu_{\phi }}/L\right){\vec{\Xi }}_{\pm }\left(\phi \right)$$

12 $$j_{\mathrm{spin}}=\left(1/4\right)\hbar \sum_{j=\pm }\int_{0}^{2\pi }\frac{d\phi }{2\pi }{\mathrm{\Xi ⃗}}_{j}^{†}\left(\phi \right)\left\{{\mathrm{\nu ̂}}_{\phi },\sigma_{z}\right\}{\mathrm{\Xi ⃗}}_{j}\left(\phi \right)$$

with {...} being the anticommutator.^75^ Here, we have defined Ξ⃗ _±_(ϕ) = χ⃗ _±_Φ­(ϕ). The velocity operator ν̂_ϕ_ = (*i*/ℏ)­[ϕ, *Ĥ*_1*d*_], defined on the basis of eq 5, contains a spin-dependent part, and it is given by
13

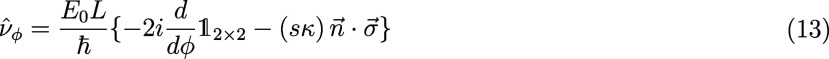




Using the latter expression, the charge current is

14 $$j_{c}^{\pm ,\kappa ,s}=\frac{e\hbar }{mL^{2}}s\left(\mathrm{l̅}\pm \frac{\kappa }{2}\right)$$

with *l̅* = ∫_0_^∞^ *dl*|*A*_*l*_|^2^ *l*. This gives a total charge current of *j*_*c*_^κ,*s*^ = (2*e*ℏ/*mL* ^2^)*s l̅*.[Fn fnb]

The difference of these currents for a given propagation direction, e.g., *s* = 1, yields (*e*ℏ/*mL* ^2^)*sκ*, which is proportional to the helicity κ, and thus changes sign upon a mirror inversion operation. The fact that this difference does not vanish indicates that the spins in the (+) and (−) states propagate with different velocities. This, in particular, allows us to define a spin polarization (SP) of the charge current:

15 $$\mathrm{SP}=\left(j_{c}^{+,\kappa ,s}-j_{c}^{-,\kappa ,s}\right)/j_{c}^{\kappa ,s}=\kappa /2\mathrm{l̅}$$

We remark that in a real system, finite-size quantization will lead to discrete values of the *l* quantum number and the integrals will become summations: ∫_0_^∞^ *dl* → ∑_*l* = 1_^∞^. The representation of the wave function Φ­(ϕ) will then read: Φ­(ϕ) = (1/√*C*)∑_*l*=–∞_^∞^ *A*_*l*_ *e* ^*ilϕ*^, with *C* = ∑_*l*=–∞_^∞^|*A*_*l*_|^2^ to ensure proper normalization.

In the special case of a single *l*-mode contributing to the summation, we can make a rough estimate of the spin polarization by assuming |*A*_*l*_|^2^ ∼ δ­(*l* – *l*_0_), so that SP = κ/(2*l*_0_), which for *l*_0_ = 1 gives a 50% polarization for κ = 1. This is of the same order of magnitude of the measured spin polarizations in, e.g., DNA.^2^

Another simple example is a Gaussian profile with standard deviation σ with $|A_{l}{|}^{2}=\left(1/\sqrt{2\pi \sigma^{2}}\right)\exp\left(-{\left(l-l_{0}\right)}^{2}/2\sigma^{2}\right)$ , which yields

16 $$\mathrm{l̅}=\int_{0}^{\infty }\mathrm{dl}|A_{l}{|}^{2}l=\frac{l_{0}}{2}\left[1+\mathrm{erf}\left(\frac{l_{0}}{\sqrt{2\sigma^{2}}}\right)\right]+\frac{\sigma }{\sqrt{2\pi }}\exp\left(-\frac{l_{0}^{2}}{2\sigma^{2}}\right)$$

For small σ, *l̅* ≈ *l*_0_ + *O*(σ^3^), so that we recover the previous result SP = κ/2*l*_0_ = 50% for *l*_0_ = 1. In the opposite case (σ ≫ *l*_0_), one gets asymptotically $\mathrm{l̅}\approx \frac{\sigma }{\sqrt{2\pi }}+\frac{l_{0}}{2}$ , which leads to $\mathrm{SP}\approx \kappa /\left(\sqrt{\frac{2}{\pi }}\sigma +l_{0}\right)\approx \kappa \sqrt{\frac{\pi }{2}}\left(1/\sigma \right)+O\left(\sigma^{-3}\right)$ . In this case, the polarization can become much smaller than 50% (the case for a single *l*-value), depending on the value of σ (see Figure 2, left panel).

**Figure 2:**
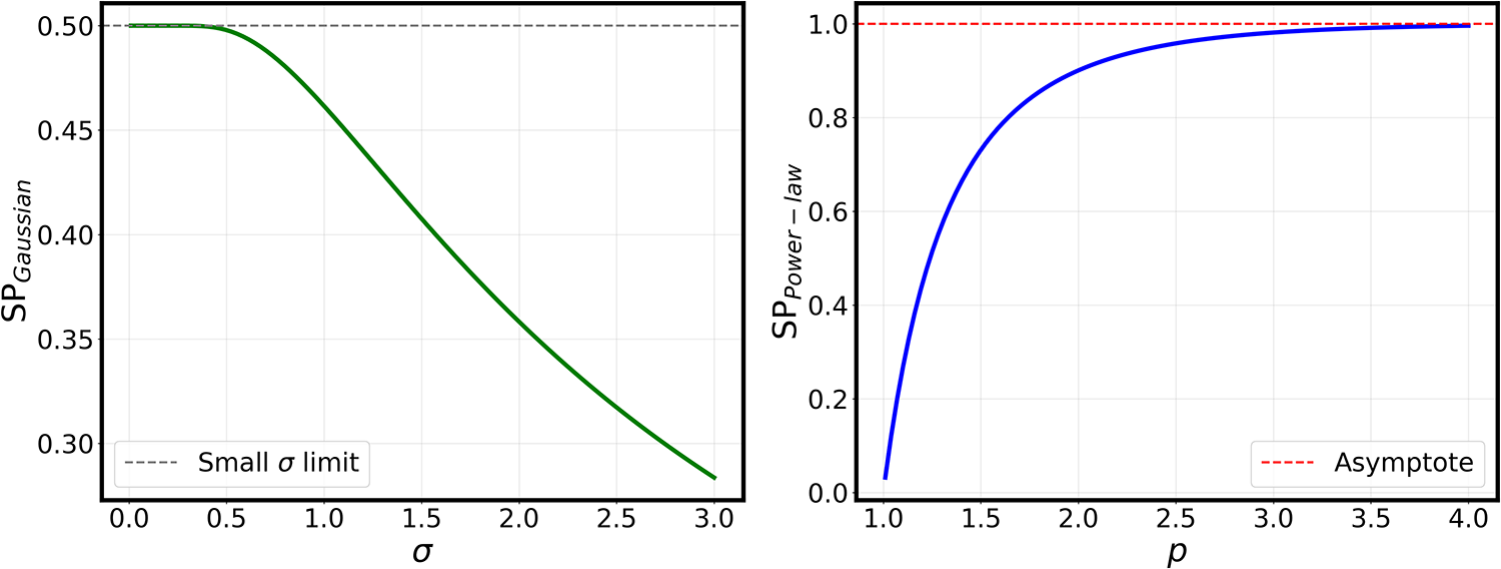
Spin polarization SP calculated according to 15 for the two assumed functions of the amplitudes *A*_*l*_. Left panel: SP for the Gaussian model as a function of the variance σ. Dashed line corresponds to the limit of small variance. Right pannel: SP for the power-law model as a function of the exponent *p*. The dashed line corresponds to the limit of large *p*.

As a last example, more appropriate for a discrete *l*-set, consider a power-law decay of the coefficients *A*_*l*_ ∝ *l* ^–*p*^. The resulting summations can be obtained in closed form in terms of Riemann’s ζ­(*p*)-function. One gets SP = *κζ*(2*p*)/2ζ­(2*p* – 1), with *p* > 1. This ratio yields already SP ≈ 77% for *p* = 1.5, and then rapidly converges to 100% for larger *p*-values (see Figure 2, right panel).

Notice, however, that in all three examples we have assumed broken time-reversal symmetry, since there would always be another mode with negative *l* yielding the opposite polarization. This can be more clearly seen from the shape of the dispersion relation, see Figure 1, and is a consequence of Kramer’s theorem.

More realistic estimates would require the formulation of a full spin transport problem with inclusion of scattering effects at, e.g., substrate-molecule interfaces, and those due to interactions with, e.g., vibrational degrees of freedom.^56,77^ We also point out that even though the magnitude of spin polarization may be different, the phenomenon we predict is present irrespective of whether the transport mechanism is ballistic or hopping.^75^ The reason is that even in the latter case the helix would create an effective spin–orbit coupling of the type we derive.

In a similar way, the spin currents can be calculated, yielding:

17 $$j_{\mathrm{spin}}^{\pm ,\kappa ,s}\left(\alpha \right)=\mp \frac{\hbar^{2}}{2\mathrm{mL}}s\cos\alpha \left(\mathrm{l̅}\pm \frac{\kappa }{2}\right)$$

which lead to the total spin current *j*_*spin*_^κ,*s*^(α) = −(ℏ^2^/2*mL*)*κs* cos α. Using the helix parameters above of DNA, we can obtain an estimate of the spin current coefficient ℏ^2^/2*mL* = 0.332 *eV nm*.

Notice that the symmetries of the spin current are *j*_spin_^±,−κ,*s*^(α) = −*j*_*spin*_^±,κ,*s*^(α), and *j*_*spin*_^±,−κ,–*s*^(α) = *j*_spin_^±,κ,*s*^(α), i.e., changing the chirality changes the sign of the spin current, while a change in chirality together with time-reversal (*s* → −*s*) leaves the spin current invariant. A nonzero spin current only emerges if time-reversal symmetry is broken; otherwise any contribution for +*s* will be canceled by a term similar to −*s*.

In a geometric picture, the chirality parameter κ should be related to the helix torsion (or to the pitch) in a suitable dimensionless quantity. It is also interesting to note that our results have a qualitative resemblance to the analytical model presented in ref 78, which, however, introduces a Rashba spin–orbit interaction in the Hamiltonian from the start. Moreover, the angle α in our case parametrizes the spin rotation vector, while in ref 78 it is related to the strength of the Rashba spin–orbit coupling.

## Conclusions

In conclusion, we have shown that a spinful particle traveling along a helical path naturally develops an effective SOC, even without an intrinsic SOC. This chirality-induced SOC (χ-SOC) is stronger than the typical relativistic SOC of light atoms, thus providing an additional source of spin polarization. Our results suggest a possible strong additional contribution to the CISS effect observed experimentally in chiral organic and inorganic materials with intrinsic helical topologies (DNA, α-helices, helicene and its derivatives, chiral crystals, etc.). In future work, it would be interesting to address issues like the temperature and length dependence of this effect, which would require a spin transport calculation, eventually including the interaction with dynamical degrees of freedom such as linear or chiral phonons. Regardless of the effect, our work suggests that geometric effects introduced by a chiral structure can generate a spin–orbit coupling contribution, which is otherwise absent in systems where mirror symmetry is not broken. It is finally worth mentioning that our model Hamiltonian can be formally adapted to different spin transport setups, including two-terminal measurements, as in break junctions,^79^ as well as more complex setups, which address a “transverse” CISS effect.^80,81^ We leave this for future studies.

## Supplementary Material



## Footnotes

a However, note that in this estimate we have used the free electron mass *m*. In a more accurate calculation, an effective mass *m** should be employed.

b Notice that for systems with a very small pitch, transport pathways along the helical axis may interfere (due to tunneling) with those following a helical path, and eventually lead to a reduction of chirality-induced effects. This is, indeed the case of helicene, as shown in ref 76 using first-principles calculations.

## Notes

Derivation of the effective 1D Hamiltonian using the confinement potential approach (PDF)
