# Supplementary material for: Chirality-Induced Spin–Orbit Coupling and Spin Selectivity
Source: J Phys Chem A. 2025 Sep 30;129(40):9504–10. doi: 10.1021/acs.jpca.5c05383 (PMC12516721; doi:10.1021/acs.jpca.5c05383)
Supplement: Supplementary file 1 [file jp5c05383_si_001.pdf]

Supplementary Information

Chirality-induced Spin-Orbit Coupling and Spin Selectivity

Massimiliano Di Ventra*
Department of Physics, University of California San Diego, La Jolla, CA, 92093, USA

Rafael Gutierrez†
Institute for Materials Science and Max Bergmann Center of Biomaterials, TU Dresden, 01062 Dresden, Germany

Gianaurelio Cuniberti‡
Institute for Materials Science and Max Bergmann Center of Biomaterials, TU Dresden, 01062 Dresden, Germany and Dresden Center for Computational Materials Science (DCMS), TU Dresden, 01062 Dresden, Germany

* diventra@physics.ucsd.edu
† rafael.gutierrez@tu-dresden.de
‡ gianaurelio.cuniberti@tu-dresden.de

Derivation of a 1-dimensional Hamiltonian on a helical pathway

Since we are only considering kinetic energy terms in the initial (spin-diagonal) Hamiltonian, our starting point is the general form of the Laplacian operator in general curvilinear coordinates (in units of $\hbar^2/(2m)$):

$$\hat{T} = \frac{1}{\sqrt{g}} \partial_n (\sqrt{g} G^{nm} \partial_m) \tag{S1}$$

Here, g is the (positive) determinant of the associated metric tensor and G its inverse. For the helical tube shown in Fig. 1 of the main text, we can choose a vector basis parametrized with the arc length s. Any neighborhood of a helical path described by a vector $\mathbf{X}$(s) can thus be generally written as: $\mathbf{R}(s) = \mathbf{X}(s) + q_1 \mathbf{N}(s) + q_2 \mathbf{B}(s)$ in terms of the unit normal vector $\mathbf{N}$(s) and binormal vector $\mathbf{B}$(s) to the helical path in a Frenet-Serret frame. The metric tensor can be obtained as $g_{nm} = \partial_n \mathbf{R} \cdot \partial_m \mathbf{R}$ with $n = s, q_1, q_2$, resulting in:

$$g_\epsilon = \begin{pmatrix} A_\epsilon^2 + \epsilon\tau^2(q_1^2 + q_2^2) & -\epsilon\tau q_2 & \epsilon\tau q_1 \\ -\epsilon\tau q_2 & \epsilon & 0 \\ \epsilon\tau q_1 & 0 & \epsilon \end{pmatrix}, \tag{S2}$$

with $A_\epsilon = \sqrt{f} = (1 - \sqrt{\epsilon}\rho q_1)^{1/2}$. Notice that this basis is non-orthogonal. The corresponding inverse G_ϵ is given by:

$$G_\epsilon = \frac{1}{A_\epsilon^4} \begin{pmatrix} 1 & \tau q_2 & -\tau q_1 \\ \tau q_2 & \frac{1}{\epsilon}[{A_\epsilon}^2 + \tau^2 q_2^2] & -\tau^2 q_1 q_2 \\ -\tau q_1 & -\tau^2 q_1 q_2 & \frac{1}{\epsilon}[{A_\epsilon}^2 + \tau^2 q_1^2] \end{pmatrix}. \tag{S3}$$

For convenience (see the subsequent developments below), we have already rescaled the transverse coordinates q_1, q_2 with a small factor $\sqrt{\epsilon}$, $q_{1,2} \to \sqrt{\epsilon} q_{1,2}$ which will control the strength of the transverse confinement potential and provide a natural expansion parameter.

Our aim is to obtain an effective 1-dimensional Hamiltonian by projecting out transversal degrees of freedom. For this, a confinement potential $V_\lambda(q_1, q_2)$ is added to the kinetic energy operator $\hat{T}$, with λ being a measure of the confinement, and the (formal) limit $\lambda \to \infty$ considered.

The specific form of the confinement potential, is in general terms, arbitrary. When applied to real molecular systems, this potential is related to the electrostatic potential distribution in the molecular frame, and it is, thus, dependent on the chemical composition. However, at the level of abstraction we are working, our choice is guided by Occam's razor, so that we assume few minimal conditions: the confinement potential should (i) be a continuous function, (ii) have a minimum on all points along the helical pathway, (iii) allow for an analytically closed solution of the transverse Schrödinger equation to be defined later, i.e., it does not depend on the arc length s, and (iv) be spin-independent.

Then, to lowest order in a Taylor expansion, similar to what is done for modelling quantum wells, we chose a harmonic confinement with SO(2) rotational symmetry (another example is briefly discussed at the end of this section): $V_\lambda(q_1, q_2) = \lambda^2(q_1^2 + q_2^2)/2 = \lambda^2 r^2/2 = \lambda^2 r^2/2\varepsilon$, where in the last step the coordinate rescaling has been introduced. Now, λ can remain finite, while the strength of the confinement is absorbed in ϵ.

For small enough, but finite, ϵ (strong confinement) the system will remain in its transverse ground state and hence, only the ground state wave functions $\Phi_0(q_1, q_2)$ of the transverse Schrödinger equation will need to be considered. As shown in Refs. [1,4,5], a mathematically clean procedure to project the full Hamiltonian on the lowest transverse states, thus leading to an effective one-dimensional problem, can be defined as:

$$\hat{H}_{eff} = \lim_{\varepsilon \to 0} \left\langle \Phi_0(q_1, q_2) | A_\varepsilon^{1/2} \hat{H} A_\varepsilon^{-1/2} - \hat{H}_\perp | \Phi_0(q_1, q_2) \right\rangle \tag{S4}$$

The term $\mathrm{H}_\perp$ is the part of the Hamiltonian including only transverse degrees of freedom −scaling as ϵ^{-1}− and which will be defined below. This procedure is more systematic than the approach used by Da Costa [2], although it leads to similar results for an infinitely strong confinement ($\epsilon \to 0$).

Based on these preliminaries, we can now use Eq. (S1) to determine $A_\varepsilon^{1/2} \hat{H} A_\varepsilon^{-1/2}$. Expanding it, we obtain:

$$\hat{H}_{eff} = A_\varepsilon^{1/2}\hat{H}A_\varepsilon^{-1/2} = \frac{1}{A_\varepsilon}\partial_n[(A_\varepsilon^2 G^{nm}\partial_m)\frac{1}{A_\varepsilon}] \tag{S5}$$

$$= G^{nm}\{\frac{1}{A_\varepsilon}(\partial_n A_\varepsilon^2)(\partial_m\frac{1}{A_\varepsilon}) + A_\varepsilon(\partial_n\partial_m\frac{1}{A_\varepsilon})\} \tag{S6}$$

$$+ A_\varepsilon(\partial_n G^{nm})(\partial_m\frac{1}{A_\varepsilon}) + \{\frac{1}{A_\varepsilon}(\partial_n A_\varepsilon)G^{nm} + (\partial_n G^{nm})\}\partial_m$$

$$+ A_\varepsilon G^{nm}(\partial_m\frac{1}{A_\varepsilon})\partial_n + G^{nm}\partial_n\partial_m + V_\epsilon(q_1,q_2).$$

A very lengthy, but straightforward calculation yields:

$$\hat{H}_{eff} = \frac{1}{A_\varepsilon^4}\{\partial_s - \tau(q_1\partial_2 - q_2\partial_1)\}^2 - \frac{1}{\epsilon}\frac{\partial_1^2 A_\varepsilon}{A_\varepsilon}$$

$$-4\frac{\partial_1 A_\varepsilon}{A_\varepsilon}\frac{\tau}{A_\varepsilon^4}q_2\{\partial_s - \tau(q_1\partial_2 - q_2\partial_1)\}$$

$$+\frac{\tau^2}{A_\varepsilon^4}\{[(\frac{\partial_1 A_\varepsilon}{A_\varepsilon})^2 - \frac{\partial_1^2 A_\varepsilon}{A_\varepsilon}]q_2^2 + \frac{\partial_1 A_\varepsilon}{A_\varepsilon}q_1\} + \frac{1}{\epsilon}(\partial_1^2+\partial_2^2) + V_\epsilon(q_1,q_2)$$

$$= \hat{T} + \frac{1}{\epsilon}(\partial_1^2+\partial_2^2) + V_\epsilon(q_1,q_2), \tag{S7}$$

from where we can identify $H_\perp$ as:

$$\hat{H}_\perp = \frac{1}{\epsilon}(\partial_1^2+\partial_2^2) + V_\epsilon(q_1,q_2). \tag{S8}$$

In the next step, we will perform an expansion in ϵ before proceeding to build matrix elements with the transverse wave functions, which can be obtained analytically by solving Eq. (S8). For the expansion, we will use the result:

$$\frac{1}{(1-\sqrt{\epsilon}C)^p} \approx 1 - p\sqrt{\epsilon}C + \frac{p(p+1)}{2}\epsilon C^2 - \frac{p(p+1)(p+2)}{6}\epsilon^{3/2}C^3 + O(\epsilon^2), \tag{S9}$$

and introduce the transverse angular momentum operator $\hat{L} = -i(q_1\partial_2 - q_2\partial_1)$. Applying this procedure, we first expand the prefactors $(A_\epsilon)^p = f_\epsilon^{p/2}$ in powers of ϵ up to order $\epsilon^{1/2}$ (we assume ϵ to be small enough, so that cutting the expansion at this order is a valid approximation):

$$\hat{T}_\epsilon = [1-2\sqrt{\epsilon}\rho q_1](\{\partial_s - i\tau\hat{L}\}^2 \tag{S10}$$

$$+\frac{\rho^2}{4}[1-\frac{3}{2}\sqrt{\epsilon}\rho q_1]]$$

$$+\sqrt{\epsilon}\{4\rho\tau[[1-\frac{5}{2}\sqrt{\epsilon}\rho q_1]q_2(\partial_s - i\tau\hat{L}) - \tau^2\rho q_1[1-3\sqrt{\epsilon}\rho q_1]\}$$

$$+\epsilon\frac{\tau^2\rho^2}{2}q_2^2[1-4\sqrt{\epsilon}\rho q_1] + O(\epsilon).$$

In the next step, we need to build matrix elements of Eq. (S10) over the ground state wave functions of the transverse Hamiltonian $H_\perp$, which, for the assumed SO(2) potential, are given by

$$|\Phi_{l,0}(\theta)>= N_l e^{il\theta}(\lambda r)^{|l|}e^{-\lambda^2 r^2/2}. \tag{S11}$$

Here, l is an angular quantum number and N_l is a normalization constant. Notice that the angular momentum operator commutes with $H_\perp$ for the SO(2) potential. Therefore, we first get to $O(\epsilon^0)$: $\left\langle\{\partial_s - i\tau L\}^2\right\rangle_0 = \{\partial_s - i\tau l\}^2$, with $\langle\cdots\rangle_0$=$\langle\Phi_{l,0}(\theta)|\cdots|\Phi_{l,0}(\theta)\rangle$.

All the other terms appearing in Eq. (S10) yield averages of the form $\left\langle q_{1,2}^2\right\rangle_0, \langle q_1 q_2\partial_{1,2}\rangle_0, \left\langle q_{1(2)}^2\partial_{2(1)}\right\rangle_0$ (up to order $\epsilon^{1/2}$). When calculating these matrix elements using $|\Phi_l(\theta)>$, one obtains contributions where the angular

momentum l is changing by $\pm1, \pm2$ (related to, e.g., integrals of the form $\int_0^{2\pi} d\theta e^{i(l-m\pm1)\theta} = 2\pi\delta_{l-m\pm1}$). Since there are no operators leading to transitions between transverse states with different angular momenta, all such contributions can be neglected, and we are left with:

$$\hat{H}_{1D}= (\partial_s - \mathrm{i}\tau l)^2 + \frac{\rho^2}{4}, \tag{S12}$$

which is, after the transformation $s = \phi/L$, the Hamiltonian of Eq. (1) in the main text.

We stress that, as a result of the previous discussion and for this specific choice of SO(2) potential, the terms involving ϵ do not contribute even for a finite ϵ. A similar analysis has been carried out in Refs. [3], and with a more rigorous mathematical presentation in Refs. [4,5]. We refer the interested reader to those articles for additional details as well as to the older study by Maraner [5].

Since the term $-i\tau l$ in Eq. (S12) appears now as a gauge field, it can be removed (for an infinite helix) by a unitary transformation. Alternatively, we could have started the analysis of Eq. (S1) by using a local orthogonal reference frame, where the metric tensor is diagonal. This can be obtained from the original frame by going to a locally rotating frame, where the normal and binormal vectors are transformed according to:

$$\begin{pmatrix} \mathbf{n} \\ \mathbf{b} \end{pmatrix} = \begin{pmatrix} \cos\vartheta(s) & \sin\vartheta(s) \\ -\sin\vartheta(s) & \cos\vartheta(s) \end{pmatrix} \begin{pmatrix} \mathbf{N} \\ \mathbf{B} \end{pmatrix} \tag{S13}$$

The angle $\vartheta(s) = \int ds'\tau(s') = \tau s$ for the case of a helical pathway with constant torsion and curvature. Using this orthogonal basis, which corresponds to a "parallel transport" frame, the term involving the angular momentum operator $\hat{L}$ does not appear, indicating that it can be removed by an appropriate change of frame. For this reason, it has not been considered in the main text, which only addresses an infinite helical system.

As mentioned above, there are in principle many possible choices of the transverse confinement with increasing complexity, e.g., anisotropic potentials, anharmonic contributions, spin-dependent potentials (in the case in which an electromagnetic field is present), etc. These different *Ansätze* deserve separate studies, which are beyond the scope of our present work.

As a second example, we address another harmonic potential, which however breaks the SO(2) symmetry, and corresponds to a square well harmonic confinement as is typical of approaches to study quantum wells. In this case, the potential energy is $V_\lambda(q_1, q_2) = (\lambda_1^2 q_1^2 + \lambda_2^2 q_2^2)/2$, and the transversal ground state wave functions are given by:

$$|\Phi_0(q_1, q_2) >= |\phi(q_1) > |\phi(q_2) >= Me^{-\lambda_1^2 q_1^2/2} e^{-\lambda_1^2 q_2^2/2}, \tag{S14}$$

where M is a global normalization constant. Performing a similar analysis as before, we get in this case (for simplicity, we take $\lambda_1 = \lambda_2$):

$$\hat{H}_{1D} = \partial_s^2 + \frac{\rho^2}{4} + 4\tau^2 g_1 + \epsilon g_2 (\frac{\rho^2}{2})^2 (1 + 6\frac{\tau^2}{\rho^2}) + O(\epsilon^{3/2}), \tag{S15}$$

with $g_1 = (\langle q_1\partial_1\rangle_0)^2 \neq 0$ and $g_2 = \langle q_1^2\rangle_0 = \langle q_2^2\rangle_0 \neq 0$. Notice that the term $O(\epsilon^1)$ is proportional to the mean square fluctuations along the transverse coordinates q_1, q_2. If only contributions $\sim \epsilon^0$ are kept, then there is, besides the curvature-dependent geometric potential, a torsion-dependent geometric potential:

$$\hat{H}_{1D} = \partial_s^2 + \frac{\rho^2}{4} + 4\tau^2 g_1 + O(\epsilon), \tag{S16}$$

but the angular momentum term is absent, since the SO(2) symmetry is broken. The term proportional to τ^2 arises from a contribution of the form $\langle q_1\partial_1 + q_2\partial_2 + 2q_1q_2\partial_1\partial_2\rangle_0$, which was originally part of $\hat{L}^2$.

As a last example, we consider a square well with infinite walls, i.e., $V(q_1, q_2) = 0$, if $|q_{1,2}| \leq \epsilon/2$ and $V(q_1, q_2) = \infty$, if $|q_{1,2}| > \epsilon$. The corresponding eigenfunctions are given by (assuming the same size of the confinement along the q_1 and q_2 directions):

$$|\Phi_{,n}0(q_1, q_2) >= |\phi_n(q_1) > |\phi_n(q_2) >= \frac{2}{\epsilon} \cos^2 \frac{\pi}{2\epsilon}(2n + 1), \tag{S17}$$

with the ground state corresponding to $|\Phi_{0,n=0}(q_1,q_2)>=\frac{2}{\epsilon}\cos^2\frac{\pi}{2\epsilon}$. Along the same lines as in the previous two examples, we have to perform first the ϵ-expansion and then carry out the projection on the ground state. The lowest order terms which survive the projection are $O(\epsilon^0)$, while the next higher order contributions are $O(\epsilon^{5/2})$ and will thus be left out:

$$\hat{H}_{1D}=\partial_s^2+\frac{\rho^2}{4}+2\tau^2\{\langle q^2\partial^2\rangle_0-\langle q\partial\rangle_0-(\langle q\partial\rangle_0)^2\}+O(\epsilon^{5/2}), \tag{S18}$$

where we have already taken advantage of the fact that, e.g., $\langle q_1\partial_1\rangle_0=\langle q_2\partial_2\rangle_0$, etc. Using $\langle q^2\partial^2\rangle_0=1-\pi^2/6$ and $\langle q\partial\rangle_0=-1$, we finally obtain :

$$\hat{H}_{1D}=\partial_s^2+\frac{\rho^2}{4}+2\tau^2(1-\frac{\pi^2}{6}), \tag{S19}$$

which shows again a torsion-dependent contribution, which is in this case negative and thus reduces the influence of the curvature-dependent term.

The different results when using the SO(2) and the square-well potentials are not unphysical: they simply reflect the fact that different confinement potentials provide different symmetries of the boundaries in the neighborhood of the helical path and this manifests in different geometric potentials. Keeping the much smaller $O(\epsilon)$ contributions in the case of the square well with harmonic confinement or the $O(\epsilon^{5/2})$ terms in the square well with infinite walls will only add further smaller position-independent corrections to the quantum geometric potentials.

We stress that all the previously obtained corrections to the geometric potential are spin-independent and, for a given helical geometry (with a given pitch and radius), they will only provide a renormalization of the energy origin in the electronic spectrum. This can be easely seen, e.g., by calculating the eigenvalues of the Hamiltonian in Eq. (5) in the main text, by including the additional corrections from Eq. (S15). The eigenvalues are given by:

$$\tilde{E}^{\kappa}_{\pm,l}=l^2\pm\kappa l+\frac{1}{4}(1-\rho R)-4\tau^2 g_1-\epsilon g_2(\frac{\rho^2}{2})^2(1+6\frac{\tau^2}{\rho^2}), \tag{S20}$$

which clearly shows that the spinor eigenvectors will be independent of ϵ, and thus the obtained results on the charge and spin currents will not be affected by this energy corrections, since only the eigenvectors are used in their calculation. Moreover, since the geometric potentials commute with the spin rotation operator introduced in the main text, they do not play a fundamental role in the subsequent discussion that follows the introduction of the geometric spin-orbit coupling in the manuscript.

References

1. Y.-L. Wang, M.-Y. Lai, F. Wang, H.-S. Zong, Y.-F. Chen, Phys, Rev. A **97**, 042108 (2018)
2. R. C. T. da Costa, Phys. Rev. A **23**, 1982 (1981).
3. K. Michaeli, R. Naaman, J. Phys. Chem C **123** 17043 (2019).
4. M. Geyer, R. Gutierrez, G. Cuniberti, J. Chem. Phys. **152**, 214105 (2020).
5. P Maraner, J. Phys. A: Math. Gen. 28, 2939 (1995).
